# 32.3 MAGNIFYING THE PROTEOME OF SCHIZOPHRENIA BRAINS

**DOI:** 10.1093/schbul/sby014.134

**Published:** 2018-04-01

**Authors:** Mariana Fioramonte, Daniel Martins-De-Souza

**Affiliations:** University of Campinas (UNICAMP)

## Abstract

**Background:**

In the post-genomic era, proteomics has emerged as a powerful tool to unravel biomarker candidates and to understand human diseases from the molecular point of view. In the last decade, our group mapped the proteome of several post-mortem brain regions and the cerebrospinal fluid from schizophrenia patients and controls to help deciphering schizophrenia’s pathobiology. These results led us to more recently focus on subproteomes and protein interaction analysis.

**Methods:**

Using ion mobility-enhanced, data-independent acquisitions and 2D-nano UPLC fractionation, we investigated the mitochondrial, nuclear and cytosolic subproteomes of the dentate nucleus and caudate nucleus as well as the protein interactome of potential protein targets to schizophrenia.

**Results:**

As previously observed, have found recurrently the differential expression energy associated proteins as well as signaling pathways associated to glutamatergic dysfunction. Proteins associated to translation machinery were also found differentially expressed, implicating spliceosome dysfunction to schizophrenia. In order to understanding this better, we investigated the protein interactome of hnRNPs by co-immunoprecipitation in the disease context.

**Discussion:**

Proteomics findings may provide an integrated picture of schizophrenia′s pathobiology and the identification of proteins associated to energy, signalling and translational pathways may trace back the origins of schizophrenia.

